# Hybrid TEVAR and REBOA procedures prior to fracture reduction and fixation in a displaced C-type thoracic spine fracture with bony contact to the aorta – A case report

**DOI:** 10.1016/j.ijscr.2025.112107

**Published:** 2025-10-25

**Authors:** Annika Ito, Felix Karl-Ludwig Klingebiel, Sandro-Michael Heining, Roland Bozalka, Hans-Christoph Pape, Michel Teuben

**Affiliations:** a Maastricht University, Maastricht, the Netherlands; b Department of Trauma Surgery, University Hospital Zurich, University of Zurich, Raemistr. 100, 8091, Zurich, Switzerland; c Harald-Tscherne Laboratory for Orthopaedic and Trauma Research, University Hospital Zurich, Zurich, Switzerland; d Department of Vascular Surgery, University Hospital Zurich (USZ), University of Zurich (UZH), CH-8091, Zurich, Switzerland

**Keywords:** Spinal injury, Aorta, TEVAR, REBOA

## Abstract

**Introduction and importance:**

Spinal injuries, particularly C-type fractures, are common in polytrauma patients. Surgical priorities are strongly influenced by the severity of concomitant injuries and cardiopulmonary stability. Sagitally displaced C-type fractures are associated with risks such as increased blood loss from damaged vessels and the potential for aortic rupture.

**Case presentation:**

This report describes a successful case of a 50-year-old male with a severe Th9 translational C-type spinal injury sustained in a motor vehicle accident. The patient presented with bilateral leg paresis and was hemodynamically stable. CT revealed direct contact to the aorta. A combined surgical strategy was used, including TEVAR for aortic protection and REBOA for hemorrhage control via the popliteal artery before surgical realignment and stabilization of the spine.

**Discussion:**

This case demonstrates a novel and safe approach combining TEVAR and REBOA to manage high-risk thoracic C-type spine fractures with direct aortic contact. This allows surgical teams to achieve spinal realignment while minimizing the risk of catastrophic aortic injury. The utilization of popliteal artery access facilitates concurrent vascular and spinal intervention in the prone position.

**Conclusions:**

Hybrid REBOA and TEVAR offers a safe strategy for managing severely displaced thoracic spinal C-type injuries if the aorta is injured or is at significant risk of secondary rupture i.e. during the repositioning maneuver. Utilizing the popliteal artery for vascular access allows for simultaneous spinal and minimally invasive vascular surgery.

**Level of evidence:**

Level V – Single case report.

## Highlights

• The combined use of REBOA and TEVAR enabled safe surgery for an unstable thoracic spine injury.
• TEVAR via popliteal access minimized the risk to the aorta during spinal fracture reduction.
• The hybrid vascular-spinal approach allows safe prone positioning despite aortic contact.
• This case demonstrates the feasibility of minimally invasive vascular repair in C-type spinal trauma.

## 1 Introduction

Spinal injuries affect up to 30 % of polytrauma patients, with C-type fractures being especially prevalent in severely injured cases [1,2]. C-type fractures are characterized by translational or rotational displacement of the spinal column [3]. These fractures are considered highly unstable due to the disruption of all three of the spinal columns: the anterior column (front half of the vertebral body and anterior ligament), the middle column (back half of the vertebral body and posterior longitudinal ligament), and the posterior column (vertebral arch, facet joints, spinous processes, and associated ligaments [4]). The severity of both spinal and concurrent injuries and subsequent cardiopulmonary stability determine surgical priorities and timing of surgery. In trauma surgery, the concept `life before limb` is well known and guides treatment decisions. In our view, this adage can be extrapolated to axial injuries as well: `life before spine` [5,6]. Despite the presence of severe spinal injuries with neurological deficits or spinal cord injuries, the priority of treatment in severe trauma with spinal involvement is to stabilize physiological function [7]. Displaced C-type injuries are associated with increased local blood loss due to disruption of para-spinal vessels [8]. Concurrent hypothermia, acidosis and coagulopathy, which are typical features of severe trauma, may result in even more profound hemorrhage [7]. Furthermore, in the specific case of concomitant aortic tears at the level of the fracture, massive blood loss and subsequent mortality may occur [9]. Direct contact of a sharp bone fragment with the descending aorta is a risk factor for subsequent iatrogenic injury and massive intra-thoracic blood loss. Mandatory reduction maneuvers in these patients to restore spinal alignment may provoke devastating iatrogenic aortic lesions. Currently, little is known about the optimal treatment of patients with severely displaced C-type fractures of the spine with direct fracture contact to the aorta and a need for operative fracture reduction and stabilization.

The authors report on the successful treatment of a C-type spine injury with sagittal fracture displacement and fracture contact to the descending aorta in a severely injured trauma patient. The aim of the current report is to highlight the novel concept of parallel minimal invasive aortic protection by TEVAR (Thoracic Endovascular Aortic Repair) installation and prophylactic REBOA (Resuscitative Endovascular Balloon Occlusion of the Aorta) placement - in prone positioning via the popliteal artery - prior to spinal fracture reduction to protect the aorta for iatrogenic and potentially fatal tears. This case report has been reported in line with the SCARE checklist [10].

## 2 Case presentation

A 50-year old male patient was referred to the trauma bay of our level one trauma center following a motor vehicle accident. The patient was a pedestrian who had been hit by a taxi travelling at approx. 60 km/h while crossing the street. He was found with bilateral leg paralysis with otherwise preserved orientation and a GCS of 15. The patient was directly transferred to our institution and admitted within 60 min after trauma. In the pre-hospital setting the patient was cardiopulmonary compensated (BP: 110/83, HR: 70, RR: 17, SpO2: 96 %) and had bruises on his chest and left shoulder and a suspected left-sided lower leg injury, which was splinted.

Pre-clinical findings were confirmed during the primary survey and as the patient remained hemodynamically stable, a spiral CT-scan was performed.

**Image 1:**
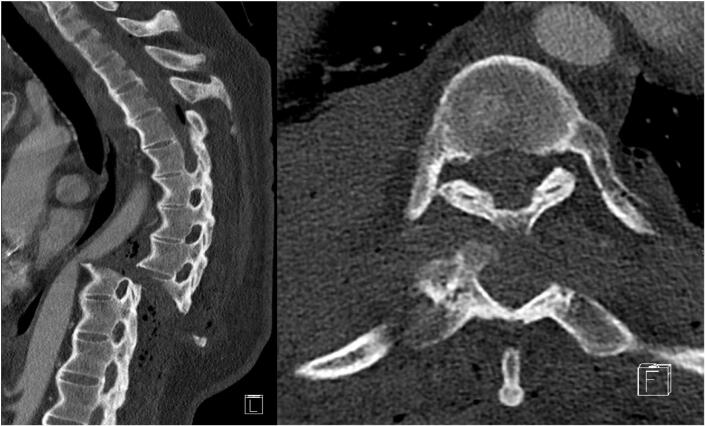
CT imaging at admission (left: sagittal, right: axial), C Type fracture with anterior fracture displacement and proximity to the aorta.

The CT-scan demonstrated a hematothorax and multiple fractured ribs. A subcapital fracture of the left humerus and a dislocated fracture of the left-sided lower leg. No intracranial hemorrhage. The neurological symptoms attributed to a translational injury of Th9 with direct contact to the aorta and transection of the spinal cord. A spike-fragment of the fractured vertebral scratched the descending aorta and caused a local arterial induration. However, an actual aortic tear or dissection was not present (Image 1). The patient also presented a distinct ankylosing spondylitis.

In line with the concepts of ATLS (Advanced Trauma Life Support), a chest tube was placed in the right hemithorax first. We further planned an emergency restoration of spinal alignment and subsequent stabilization of the spine in this cardiopulmonary stable patient. Emergency fracture fixation of the lower leg fracture had lower priority and was planned after spinal surgery by an external fixator (plus dermatofasciotomy if mandated) in the case of persistent systemic stability of the patient.

To fully restore spinal alignment, a reduction of spinal fracture fragments over a distance of 5 cm was required in the sagittal plane. Given the bony prominence in adherence of the descending aorta, there was a high chance of causing iatrogenic aortic tears and potential massive blood loss. Therefore, we decided to attempt shielding the descending aorta at the level of the fracture. In order to improve efficiency and to allow for later intra-operative vascular interventions for hemorrhage control (REBOA) we decided to obtain vascular access in prone positioning via the popliteal artery of the unaffected right leg.

**Image 2:**
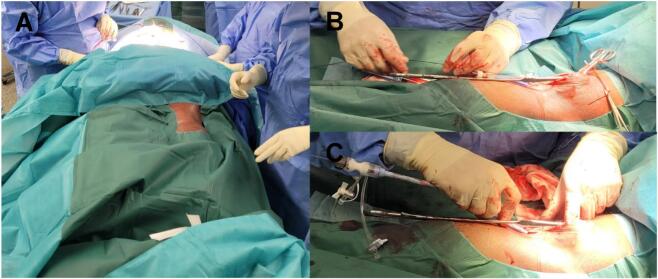
Intraoperative images displaying prone patient positioning, allowing simultaneous spinal and endovascular intervention (A) and dorsal approach to the popliteal artery on the right side for catheter introduction (B—C).

Spinal and vascular approaches were performed simultaneously: the patient was transferred to the operating room and turned *en-block* into prone position. Spinal alignment was determined by fluoroscopy. Although no profound fracture reduction was observed. Then, a cut-down in the right popliteal fossa and exposure of the first segment of the popliteal artery was performed. A small caliber popliteal artery (5 mm wide) was encountered (Image 2).

**Image 3:**
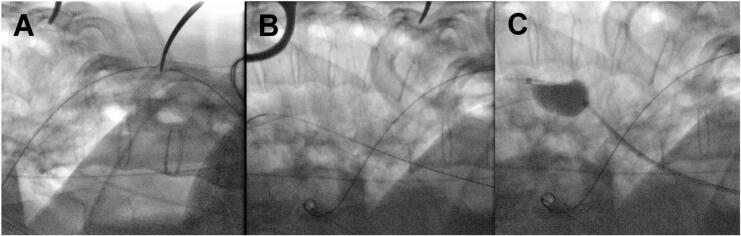
Serial intraoperative fluoroscopy images demonstrating guide wire positioning above fracture level and REBOA inflation (test) (A-C).

Puncture of the popliteal artery was performed for the placement of a 12 F sheath to allow for REBOA-installment. Replacing the wire on Terumo. Insertion of a pigtail catheter. Repeated angiography allowed for proper identification of the fracture-related aortic induration and exclusion of aortic bleeding. Introduction of the REBOA-system. Intra-operative testing by inflation and deflation of the balloon under fluoroscopic control (Image 3).

**Image 4:**
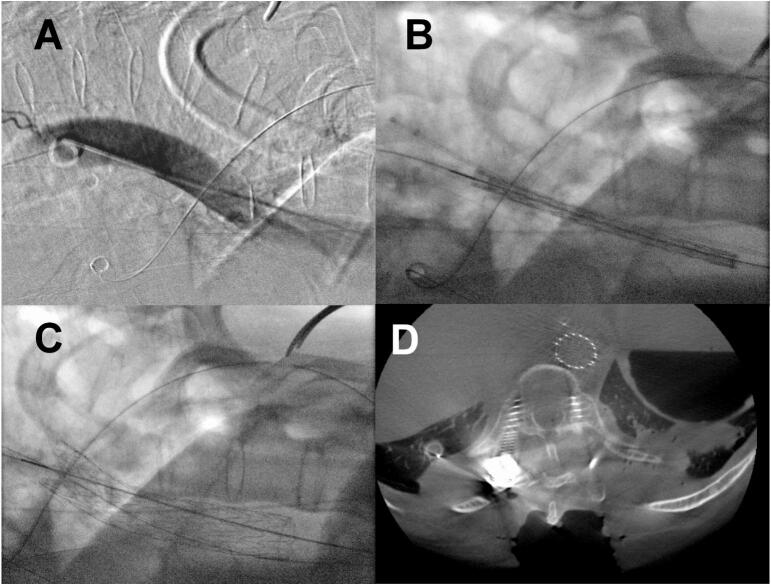
Serial intraoperative fluoroscopy images demonstrating TEVAR positioning and placement (A-C), intraoperative 3D O-arm scan with confirmation of adequate TEVAR placement.

This ensured easy REBOA positioning in case of massive blood loss. Then, removal of the REBOA system. Introduction of a Lunderquist wire and replacement of the sheath to a Dryseal sheath 22 Fr. Thereafter a GORE 26x100mm stent-graft prosthesis was inserted. The distal end of the stent prosthesis is located at the level between the 11th and 12th thoracic vertebrae, i.e. above the exit of the coeliac trunk (Image 4).

**Image 5:**
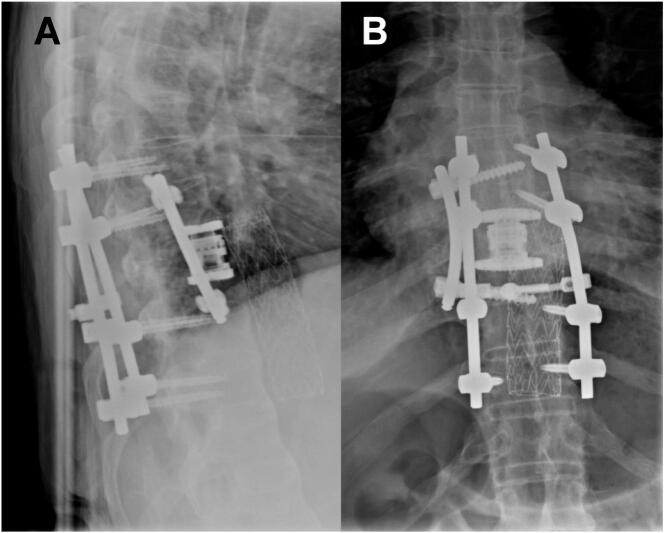
Three months postoperative imaging in upright position demonstrating adequate status of cage and instrumentation (A: lateral view, B: posterior/anterior view).

Due to the fracture related anatomical alterations and strong resistance, it was not possible to further introduce the stent prosthesis. Control angiography shows an unfolded prosthesis and a good capillarisation of the acra of the right foot. The surgical wound is left open for the time being to allow for emergency re-installment of the REBOA-System. In parallel, spinal dorsal exploration occurred. No reduction maneuvers were performed prior to the completion of the previously described vascular intervention. Afterwards, a controlled fluoroscopy guided reduction of the spinal fracture could be safely performed with following open dorsal spondylodesis of Th7/8 to Th10/11 and subsequent laminectomy. The patient's coagulopathic state (INR: 1.4, PT: 14.7, aPTT: 56, Thrombocyte count: 55 g/l, Fibrinogen: 1.0 g/l) and invasiveness of the intervention resulted in over 1500 ml blood loss and subsequent need for circulatory support with high-dose catecholamines. Subsequently, suboptimal screw placement was tolerated. After four days, corpectomy of Th9 with cage-implantation an additional lateral screw-rod fixation was performed by an anterior approach to support the short-segment dorsal construct. Image 5 displays three-month follow-up imaging. Of note, there were no complications from the concurrent extremity injuries, and adequate fracture healing was achieved with humeral plating and tibial nailing.

One year after the trauma, the patient continues to experience complete paraplegia below the Th8 vertebra. The hardware has remained stable and there have been no clinical or radiographical abnormal findings. However, the patient has shown a high level of adaptability to this condition and is now able to move around with the assistance of a wheelchair. Furthermore, he has been able to maintain his previous employment on a 50 % time basis.

## 3 Discussion

Polytrauma patients requiring emergency surgery for displaced C-type spinal fractures pose significant challenges for trauma surgeons. During spinal surgery, the trauma triad of death factors (acidosis, hypothermia and coagulopathy) may worsen, and secondary bleeding from other injuries can occur [7]. Patient positioning (e.g. prone or lateral) during surgery complicates the ability to perform lifesaving procedures for intra-thoracic or abdominal hemorrhage. Surgeons must be aware of these risks and prepare for intraoperative emergencies, making preoperative planning crucial even with limited time. Collaboration with anesthesia and other surgical specialties (e.g., abdominal, thoracic, or vascular) is essential for planning bail-out strategies [11,12].

In the current case, there were no concomitant injuries that could have caused ongoing blood loss prior to the start of spinal surgery. However, the proximity of a fracture fragment to the aorta increased the risk of aortic rupture during fracture reduction. Similar fatal cases have been documented in previous literature [13]. Advances in minimally invasive intraoperative bleeding control facilitated preventive measures. We planned a combined procedure in collaboration with vascular surgeons using REBOA (Image 3) for optimizing the haemodynamic status during potential aortic rupture and TEVAR (Image 4) to protect the aorta at the fracture site during manipulation. The patient was positioned in the prone position, and we tested the REBOA system prior to the spinal intervention, allowing for repositioning if necessary. Fortunately, the TEVAR procedure was uneventful, and there was no need to activate REBOA. In contrast to Zhao et al., the REBOA-device has been introduced in prone position, rather than shifting the patient with a catheter in place [11]. Given the highly unstable nature observed within the overall fracture pattern, a subsequent procedure was deemed necessary to increase anterior column stability. This procedure entailed a corpectomy and the implantation of a cage.

Cultera et al. and Matsuo et al. also report similar cases in which prophylactic TEVAR was performed prior to spinal surgery due to the close proximity of the fracture fragment to the aorta, posing a significant risk of aortic injury during the spinal procedure [14,15]. In both cases, TEVAR was conducted via femoral artery access, which either required repositioning the patient for subsequent posterior spinal instrumentation or necessitated delaying the spinal surgery altogether. The importance of prioritizing vascular stabilization before spinal intervention is further supported by the case series of Santoro et al., which found that 60 % of patients who underwent spinal surgery prior to vascular repair did not survive [16].

Of note, TEVAR placement further minimized local blood loss from the segmental artery, but potentially blocks the course of other relevant arteries in the case of more caudal placement (cave: solid or hollow-organ ischemia). In order to avoid leg ischemia or complications (pseudoaneurysm, embolism), we chose arterial access via the uninjured leg. Other indications for choosing the contralateral limb for vascular access were previous vascular interventions or suboptimal distal drainage (<2 arteries on the lower leg). Therefore, we suggest performing a CT-Angio of both legs prior to the start of surgery to determine the vascular status especially in older patients or those with relevant comorbidities.

Although this case presents the prophylactic use of hybrid TEVAR and REBOA procedures, we do not propose this approach as a standard treatment for all dislocated spinal fractures. The necessity of such prophylactic measures should be determined on a case-by-case basis. Preemptive endovascular interventions may be considered to reduce intraoperative risks when spinal realignment presents a potential threat of vascular injury.

## 4 Conclusion

The combination of hybrid REBOA (Resuscitative Endovascular Balloon Occlusion of the Aorta) and TEVAR (Thoracic Endovascular Aneurysm Repair) offers a safe strategy for managing severely displaced thoracic spinal C-type injuries when the aorta is injured or is at significant risk of secondary rupture. Spinal and minimally invasive vascular surgery can be performed simultaneously by obtaining vascular access via the popliteal artery.

## Consent

Written approval to publish this case report was obtained from the patient.

## Ethical approval

An ethical approval was not required.

## Funding

External funding was not required.

## Author contribution

A.I.: Data curation, Investigation, Methodology, Writing – original draft, Writing – review & editing.

F.K.: Formal analysis, Investigation, Methodology, Image creaton, Writing – original draft, Writing – review & editing.

S.H.: Investigation, Writing – review & editing.

R.B.: Investigation, Writing – review & editing.

HC.P.: Conceptualization, Formal analysis, Investigation, Methodology, Project administration, Supervision, Writing – original draft, Writing – review & editing.

M.T.: Conceptualization, Data curation, Formal analysis, Investigation, Methodology, Project administration, Supervision, Writing – original draft, Writing – review & editing.

## Guarantor

Michel Teuben.

## Research registration number

No clinical study was performed.

## Conflict of interest statement

On behalf of all the authors, the corresponding author states that there are no conflicts of interest. No external funds were used for this research project.
